# Is Perceived Family Cohesion Associated with Family Caregiver Role and Caregiver-Related Characteristics? A Comparison of Spousal and Adult-Child Caregivers

**DOI:** 10.3390/healthcare14040472

**Published:** 2026-02-13

**Authors:** Jie Huang, Xinjie Zhou, Jun Yao, Li Zhang

**Affiliations:** 1 School of Health Policy and Management, Nanjing Medical University, Nanjing 211166, China; huangjie@stu.njmu.edu.cn (J.H.); zhouxinjie@stu.njmu.edu.cn (X.Z.); 2 School of Health Economics and Management, Nanjing University of Chinese Medicine, Nanjing 210023, China

**Keywords:** informal caregiver, family cohesion, dementia care, older adults

## Abstract

**Highlights:**

**What are the main findings?**

- Adult-child caregivers reported higher family cohesion than spousal caregivers.
- Caregiver-related characteristics mediate caregiver role and family cohesion.

**What are the implications of the main findings?**

- Interventions that strengthen caregivers’ physical health, social support, self-efficacy, and family involvement in caregiving can equalize the perception of family cohesion between spousal caregivers and adult-child caregivers.

**Abstract:**

**Background:** Spousal caregivers and adult-child caregivers are the main sources of informal care of patients with dementia. They are primarily responsible for providing daily assistance, emotional support, and long-term care within the family. **Methods:** A cross-sectional survey was conducted in June 2024 in Nanjing, Jiangsu Province, China. A total of 410 family caregivers (154 spousal and 256 adult-child caregivers) participated. Perceived family cohesion was measured using the Family Adaptability Cohesion Scale, Second Edition, Chinese version (FACES II-CV). Caregiver-related characteristics included the following: caregivers’ self-reported physical health, social support, self-efficacy, and the number of family members assisting in caregiving. **Results:** (1) adult-child caregivers reported significantly higher perceived family cohesion than spousal caregivers (*p* < 0.001); (2) regression analyses revealed that caregiver role and caregiver-related characteristics were positively associated with perceived family cohesion (all *p* < 0.05); and (3) mediation analyses revealed that caregiver-related characteristics mediate the relationship between caregiver role and perceived family cohesion, with mediating effects of 0.109, 0.293, 0.087 and 0.174, respectively. **Conclusions:** Given that caregiver-related characteristics act as mediators of family cohesion, interventions should focus on strengthening caregivers’ physical health, social support, self-efficacy, and family involvement in caregiving to equalize the perception of family cohesion between spousal caregivers and adult-child caregivers.

## 1. Introduction

China has the highest number of people with dementia worldwide, with the number of people with dementia accounting for approximately one-quarter of the global number of people with dementia [1]. A nationally representative study shows that among people aged 65 years and older in China, the prevalence of dementia was 5.14% (95%CI: 4.71–5.57) in 2014 [2] and 5.60% (95%CI: 3.50–7.60) in 2019 [3]. In China, the majority of people with dementia continue to be cared for at home, primarily by family members, which is likely due to limited access to professional care services [4]. Dementia significantly affects not only people with dementia but also their family caregivers. The burden of caring for people with dementia is often greater than for most other disabilities, impairments, or illnesses [5]. Many family caregivers experience varying degrees of adverse psychological states, such as anxiety and depression, which can negatively impact their mental health [6].

Spousal caregivers and adult-child caregivers constitute the primary groups of family caregivers. They are primarily responsible for providing daily assistance, emotional support, and long-term care within the family [7]. While research has demonstrated that these two groups may experience distinct caregiving outcomes when supporting a person with dementia, it remains unclear which group faces greater challenges. Compared to adult-child caregivers, spousal caregivers often exhibit poorer health outcomes largely due to their older age [8]. Moreover, spousal caregivers are more likely to live with the patient and provide care over extended periods [9]. As a result, spousal caregivers are more likely to experience a heavier caregiving burden and worse mental health outcomes [10]. In contrast, a distinguishing feature of adult-child caregivers is their tendency to actively seek information and social support. For example, when it comes to medical decision-making, more adult-child caregivers turn to healthcare professionals as well as friends and family for guidance and information [11]. Adult-child caregivers, although in better physical condition, often juggle caregiving responsibilities with employment, childcare, and other family obligations, leading to heightened role conflict [12]. However, it has also been suggested that these additional roles may, in some cases, complement or even enhance their caregiving responsibilities [13].

Family cohesion refers to the emotional connectedness among family members, encompassing mutual support, warmth, and care. It is widely recognized as a key indicator of overall family functioning [14]. Unlike commonly examined outcomes such as caregiver burden or mental health, which capture individual stress responses, perceived family cohesion represents a family-level process that shapes caregiving cooperation, role negotiation, and long-term sustainability of care. According to family systems theory, there are variations in the perception of the family by different members [15]. Most existing research on perceived family cohesion has focused on parent–child relationships. For example, Ohannessian [16] found that adolescents and their mothers often differ in their perceptions of family cohesion. American parents reported higher levels of family cohesion than their adolescent children, although no significant differences were observed in perceived family conflict [17]. Spousal and adult-child caregivers, as different family members, may also differ in their perceptions of family cohesion. Spousal caregivers typically form a dyadic caregiving relationship characterized by high emotional interdependence, cohabitation, and long-term care provision. As dementia progresses and marital reciprocity diminishes, spouses are more likely to perceive disruptions in family cohesion. In contrast, adult-child caregivers rely more heavily on communication, coordination, and support among family members. Consequently, they tend to view family cohesion primarily as a collective caregiving resource rather than solely as an emotionally intimate relationship [18,19]. Examining these differences may provide clinically meaningful insights for family-centered dementia care interventions by informing role-specific support strategies.

In addition to the caregiver role, numerous studies have demonstrated that caregiver-related characteristics are also associated with perceived family cohesion. One effective approach to reducing caregiver burden is to identify and address the unmet needs of family caregivers by providing appropriate support [20]. Therefore, family caregivers’ needs should be considered a primary factor. A family’s ability to cope with a crisis largely depends on the availability of resources. When resources are insufficient, family dysfunction may result [21]. At the individual level, family caregivers often report the need for personal time and improved health [22]. Economic status is also strongly associated with family functioning [23]. Many Filipino family caregivers of children with cancer have been found to lack sufficient financial resources [21]. Family caregivers frequently express the need for emotional and practical support from family members, friends, and healthcare professionals [24]. Family resources, such as emotional closeness among family members, are also key to maintaining cohesion [25]. Spousal caregivers receive less support from other family members compared to adult-child caregivers [10]. Furthermore, social support is positively associated with family functioning [26]. For example, higher levels of social support for caregivers of children with autism spectrum disorders are associated with greater perceived family cohesion, which in turn can improve their quality of family life [27].

Another important caregiver-related characteristic is how caregivers perceive and respond to stressful events. Self-efficacy refers to an individual’s self-regulatory capacity to manage challenges and reflects one’s judgment of their ability and confidence to achieve specific goals [28]. Multiple studies have confirmed a negative correlation between self-efficacy and perceived stress. Individuals with higher self-efficacy typically report lower levels of stress [29]. Self-efficacy plays an important role in caregivers’ ability to cope with stress. In a study of caregivers of elderly patients with multiple chronic conditions, higher self-efficacy was associated with reduced caregiver stress and improved sleep quality [30]. Another study also highlighted the importance of self-efficacy in alleviating caregiver burden and enhancing patients’ quality of life [31]. In addition, self-efficacy has been positively associated with family functioning. That is, family caregivers with higher self-efficacy tend to report healthier family functioning [32].

**Figure 1:**
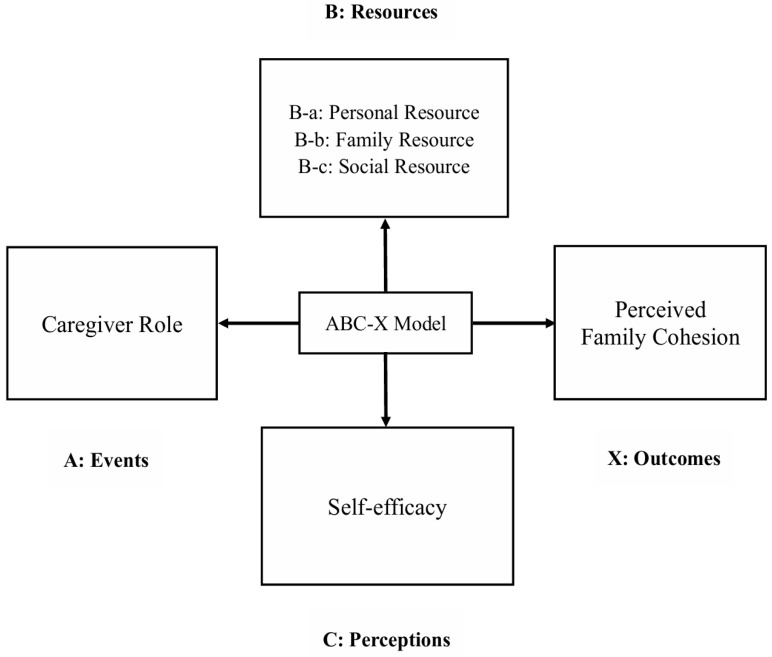
Theoretical model of factors associated with perceived family cohesion.

Hill’s ABC-X model [33] served as the theoretical framework for this study. This model is commonly used to explore and explain how families adapt to and cope with stressful and complex situations. The ABC-X model consists of factors A, B, C, and X, allowing for a theoretically grounded examination of how caregiver role and caregiver-related characteristics jointly shape outcomes of family cohesion. The model has been widely applied in studies of family caregivers [34,35]. Factor A refers to events that can cause changes in the family system; Factor B refers to available coping resources, including personal, family, and social resources; Factor C represents perceptions and interpretations of the stressful event; and Factor X is the outcome resulting from the interaction of A, B, and C, reflecting the evaluation of family functioning, which aligns directly with perceived family cohesion as the primary outcome of this study. As shown in Figure 1, in the present study, Factor A was defined as the caregiving role undertaken by different family caregivers while Factor B and Factor C together represent the caregiver-related characteristics. Specifically, Factor B was defined as caregivers’ resources, including personal, family, and social resources. Factor C was defined as caregivers’ self-efficacy, an indicator reflecting their perceived capacity to manage care demands and make effective decisions, making it a conceptually appropriate proxy for cognitive appraisal in this context. This operationalization is consistent with previous studies employing the ABC-X framework [35]. Factor X was defined as perceived family cohesion.

Based on the ABC-X model, this study had three specific objectives:

O1: To examine whether perceived family cohesion differs by caregiver role (spousal vs. adult-child caregivers).
O2: To investigate the associations between caregiver-related characteristics and perceived family cohesion.
O3: To test whether caregiver-related characteristics mediate the relationship between caregiver role and perceived family cohesion.

Accordingly, the following hypotheses were proposed:

H1: Caregiver role is significantly associated with perceived family cohesion.
H2: Caregiver-related characteristics are significantly associated with perceived family cohesion.
H3: Caregiver-related characteristics mediate the relationship between caregiver role and perceived family cohesion.

To address these objectives, we constructed a regression model with perceived family cohesion as the dependent variable and caregiver role, along with caregiver-related characteristics as independent variables. Additionally, a mediation model was developed, in which perceived family cohesion served as the dependent variable, caregiver role as the independent variable, and caregiver-related characteristics as the mediator, in order to elucidate the mechanisms underlying their associations. This research may provide evidence for implementing interventions targeting caregiver-related characteristics, potentially enhancing cooperative caregiving and sustaining family functioning. The findings may also inform the development of family-based dementia care policies and services, guiding resource allocation to ensure both caregivers and patients receive optimal care outcomes.

## 2. Materials and Methods

### 2.1. Participants and Procedure

This study was conducted in June 2024 in Nanjing, Jiangsu Province, China, in collaboration with a local dementia service organization. The organization maintains a registry of patients with dementia and their primary family caregivers. Eligible participants were identified from this registry according to the inclusion and exclusion criteria. Recruitment was conducted with the support of the organization’s staff. Staff initially contacted potential participants during routine service visits to explain the study and invite participation. For those who agreed, home visits were scheduled. During the visit, trained interviewers thoroughly explained the study’s purpose, content, and key questions to the caregivers, assuring confidentiality, and obtained signed informed consent. Agency staff accompanied interviewers to facilitate communication and provide support. Ethical considerations were carefully addressed. To ensure privacy, interviews were conducted in a quiet, private area of the home whenever possible. Should other family members be present, the interview time had to be rescheduled. The interview was administered using a paper-based questionnaire, which the interviewer read aloud question by question. Caregivers responded verbally, while the interviewer recorded their answers. This approach allowed for immediate clarification and elaboration on any unclear aspects of the survey.

Inclusion criteria for patients required a formal diagnosis of Alzheimer’s disease confirmed by a medical institution, possession of a diagnostic certificate, home residence for at least six months following the diagnosis, and age 60 years or older. These criteria ensured that participants were experiencing a stable caregiving context, allowing for meaningful assessment of family cohesion. The caregivers in this study were the primary family caregivers, operationalized as the family member who assumed the principal responsibility for daily caregiving tasks, including assistance with activities of daily living, emotional support, and coordination of medical care. Caregivers were considered primary if they were the individual most consistently involved in providing care daily, as confirmed by both the patient’s medical record and family report. Inclusion criteria for caregivers were as follows: (1) aged 18 years or older; (2) immediate family members of the patient; (3) sufficient communication ability to understand and complete the questionnaire accurately; and (4) voluntary participation with signed informed consent. Exclusion criteria included: (1) caregiving duration was less than six months; (2) being a paid caregiver or babysitter; or (3) being unable to participate in a standard interview. These criteria were chosen to ensure that participants were long-term, primary family caregivers who directly experience and influence family cohesion, thereby enabling meaningful and valid comparisons between spousal and adult-child caregivers. Ultimately, a total of 410 family caregivers participated, including 154 spousal caregivers and 256 adult-child caregivers.

### 2.2. Measures

The caregiver role was analyzed as an independent variable. Based on the questionnaire item: “What is your relationship to the patient? (0 = spouse, 1 = adult-child)”, caregivers were identified as the patient’s spouse or adult-child. Only legally married spouses and biological children of the patient were included in the respective categories.

Perceived family cohesion was measured using the Family Adaptability Cohesion Scale, Second Edition, Chinese version (FACES II-CV), developed by Fei et al. [36]. The scale includes two subscales: family cohesion and family adaptability. In this study, only the family cohesion subscale was utilized. Family cohesion refers to the degree of emotional connection among family members. The subscale consists of 16 items, each rated on a 5-point Likert scale ranging from 1 (“never”) to 5 (“always”). Higher scores indicate stronger emotional connectedness among family members. Based on total scores, family cohesion is classified into four categories: disengaged (<55.9 points), separated (55.9–63.9 points), connected (63.9–71.9 points), and enmeshed (>71.9 points). FACES II-CV has been validated for use in the Chinese population and has good validity [37]. In this study, the FACES II-CV demonstrated excellent internal consistency (Cronbach’s α = 0.861).

Caregiver-related characteristics were conceptualized as mediating variables. The first dimension of caregiver-related characteristics was caregivers’ resources (Factor B). Personal resource was defined as the caregiver’s self-reported physical health (Factor B-a). This was assessed using the questionnaire item: “What do you currently consider to be your physical health status?”. Responses were rated on a 5-point Likert scale, ranging from 1 (“very unhealthy”) to 5 (“very healthy”).

Family resource was defined as the number of family members caring for the patient assisting the primary caregiver (factor B-b). This was assessed using the questionnaire item: “How many other family members assist you in caring for the patient?”. While this approach does not capture qualitative aspects such as frequency, intensity, or perceived adequacy of assistance, it provides a straightforward and measurable indicator of support within the family context.

Social resource was defined as the caregiver’s level of social support (Factor B-c), which was assessed using the Social Support Rating Scale (SSRS) developed by Xiao [38]. The scale comprises 10 items across three dimensions: subjective support, objective support, and utilization of support. The total score ranges from 0 to 66, with scores below 22 indicating low social support, 23–44 indicating moderate support, and 45–66 points indicating high support. In this study, the SSRS demonstrated excellent internal consistency (Cronbach’s α = 0.818).

Another caregiver-related characteristic is caregivers’ self-efficacy (Factor C). Self-efficacy was measured using the General Self-Efficacy Scale (GSES), originally developed by Schwarzer et al. [39] and adapted into Chinese by Wang et al. [40]. The scale contains 10 items rated on a 4-point Likert scale ranging from 1 (“not at all true”) to 4 (“completely true”). Total scores range from 10 to 40, with scores of 30–40 indicating high self-efficacy, 20–29 indicating moderate self-efficacy, and 10–19 indicating low self-efficacy. In this study, the GSES demonstrated excellent internal consistency (Cronbach’s α = 0.963).

Based on prior empirical evidence and theoretical relevance to caregiving experiences and family functioning in dementia care [41,42], the following background characteristics were collected and treated as covariates: caregiver age, sex, employment status, daily caregiving hours, years since the patient diagnosis, and whether the caregiver was contracted with a family doctor. These variables capture key demographic factors, caregiving intensity, disease-related context, and access to primary healthcare services.

### 2.3. Data Analysis

All statistical analyses were performed using SPSS version 26.0, with a significance level set at 0.05 (two-tailed). Descriptive statistics were used to summarize participant background characteristics. Differences in perceived family cohesion between spousal caregivers and adult-child caregivers were examined using independent samples t-tests and chi-square (χ2) tests. Perceived family cohesion was treated as the dependent variable in a multiple linear regression model, with caregiver role and caregiver-related characteristics included as independent variables. Caregiver age, gender, employment status, daily caregiving hours, years since patient diagnosis, and family doctor contract status were incorporated as covariates. Prior to conducting regression analyses, we examined key assumptions of linear regression, including normality of residuals, linearity, homoscedasticity, and multicollinearity. Finally, mediation analyses were conducted using the SPSS PROCESS macro version 4.0 (Model 4) to examine whether caregiver-related characteristics mediated the relationship between caregiver role and perceived family cohesion. Bootstrapping with 5000 samples was employed, and mediation effects were considered statistically significant if the 95% bootstrap confidence interval did not include zero.

## 3. Results

### 3.1. Background Information of Participants

As shown in Table 1, spousal caregivers were older than adult-child caregivers among the included family caregivers. Half (50.00%) of the spousal caregivers were aged 80 years or older, whereas adult-child caregivers were predominantly aged 60–79 years (51.95%). Regarding gender distribution, there was an equal split between males and females. However, spousal caregivers were more likely to be female (60.39%), while adult-child caregivers were more likely to be male (57.81%). The majority of caregivers were not employed (82.71%), indicating unemployment or retirement. Among adult-child caregivers, 28.91% were still working. Spousal caregivers spent more hours per day caring for the patient compared to adult-child caregivers, with the vast majority (79.87%) providing care for 20 h or more daily, likely due to cohabitation. No significant difference was observed between spousal and adult-child caregivers in terms of years since the patient diagnosis, with half (52.68%) having less than five years since onset. Similarly, only a small proportion (5.61%) of families reported having a family doctor.

### 3.2. Perceived Family Cohesion Between Spousal Caregivers and Adult-Child Caregivers

Table 2 presents differences in perceived family cohesion between spousal caregivers and adult-child caregivers. Adult-child caregivers reported significantly higher perceived family cohesion scores than spousal caregivers (*p* < 0.001). Based on the family cohesion scores, family cohesion was classified into four types: disengaged, separated, connected, and enmeshed. Among spousal caregivers, the most common type was disengaged (33.12%), followed by separated (24.68%), connected (22.73%), and enmeshed (19.48%). In contrast, among adult-child caregivers, the connected type was most prevalent (31.64%), followed by enmeshed (30.47%), separated (21.09%), and disengaged (16.80%). A chi-square test revealed a significant difference in the distribution of family cohesion types between the two caregiver groups (*p* < 0.001). These findings support H1.

### 3.3. Results of Linear Regression Models

Table 3 presents the results of three linear regression models. All variance inflation factors (VIFs) for the independent variables ranged between 1 and 5, indicating no serious multicollinearity. These models satisfy the assumptions of linear regression models. Dichotomous independent variables were recoded as dummy variables. Both unstandardized (B) and standardized (β) coefficients are presented. For interpretation of the relative strength of predictors, we focus on the standardized coefficients (β). In Model 1, the caregiver role was positively associated with perceived family cohesion (β = 0.238, *p* < 0.001), indicating that adult-child caregivers reported higher family cohesion compared to spousal caregivers. Model 1 represents the unadjusted association between caregiver role and perceived family cohesion. In Model 2, after adjusting for caregiver background characteristics, the association between caregiver role and perceived family cohesion remained significant and slightly increased (β = 0.299, *p* < 0.001), indicating that the relationship persisted after controlling for these background characteristics. The results of Models 1 and 2 again provide support for H1. Model 3 further included caregiver-related characteristics, including caregivers’ self-reported physical health, social support, self-efficacy, and the number of family members assisting in caregiving. All of these factors were significantly and positively associated with perceived family cohesion (*p* < 0.05), thereby supporting H2, which posits that caregiver-related characteristics (caregivers’ resources, self-efficacy) are associated with perceived family cohesion. The weakened relationship between caregiver role and perceived family cohesion (β = 0.138, *p* < 0.05) indicates that caregiver-related characteristics partially mediate the association between the caregiver role and perceived family cohesion.

### 3.4. Results of Mediating Effect Analyses

To examine the mediating role of different caregiver-related characteristics in the association between caregiver role and perceived family cohesion, caregivers’ self-reported physical health, social support, self-efficacy, and the number of family members assisting in caregiving were tested separately (Table 4). The results showed that all caregiver-related characteristics were significantly associated with the caregiver role. Compared to spousal caregivers, adult-child caregivers reported better physical health (β = 0.613, *p* < 0.001), higher levels of social support (β = 0.652, *p* < 0.001), and a greater number of family members assisting in caregiving (β = 0.882, *p* < 0.001). These findings suggest that adult-child caregivers have more resources, including personal, family, and social resources, when coping with family caregiver status. Additionally, adult-child caregivers reported significantly higher levels of self-efficacy compared to spousal caregivers (β = 0.429, *p* < 0.001), indicating greater confidence and perceived competence in managing caregiving-related stress.

The mediating effects of caregiver role and perceived family cohesion are presented in Table 5. Caregiver-related characteristics were found to mediate this relationship through four significant indirect pathways: (1) path1: caregiver role → self-reported physical health → perceived family cohesion (β = 0.109, 95% CI = 0.048, 0.182), (2) path2: caregiver role → number of family members assisting in caregiving → perceived family cohesion (β = 0.174, 95% CI = 0.082, 0.277), (3) path3: caregiver role → social support → perceived family cohesion (β = 0.293, 95% CI = 0.200, 0.391), and (4) path4: caregiver role → self-efficacy → perceived family cohesion (β = 0.087, 95% CI = 0.034, 0.154). The proportions of the total effect accounted for by each indirect pathway were 22.2%, 35.4%, 59.7%, and 17.7%, respectively. Since none of the 95% confidence intervals included zero, all four mediation pathways were statistically significant. These findings suggest that caregiver-related characteristics (caregivers’ resources and self-efficacy) positively mediate the association between caregiver role and perceived family cohesion. Caregivers with greater resources or higher self-efficacy tend to report higher levels of perceived family cohesion. Therefore, H3 is supported.

## 4. Discussion

The primary aim of this study was to examine whether perceived family cohesion differs by caregiver role. Adult-child caregivers reported significantly higher levels of perceived family cohesion compared to spousal caregivers. Guided by the ABC-X model, linear regression analyses demonstrated that caregivers’ resources (Factor B, including caregivers’ self-reported physical health, social support, and the number of family members assisting in caregiving) and caregivers’ self-efficacy (Factor C) were significantly associated with perceived family cohesion. A series of mediation analyses further revealed that these caregiver-related characteristics mediate the relationship between caregiver role and perceived family cohesion. These findings suggest that greater caregiver resources and higher self-efficacy are associated with stronger perceptions of family cohesion.

First, to examine whether perceived family cohesion differs by caregiver role, we compared the family cohesion scores between spousal caregivers and adult-child caregivers. Results showed that adult-child caregivers reported significantly higher levels of family cohesion than spousal caregivers, with the most frequently reported category being the connected type (31.64%). This finding indicates an association between caregiver role and perceived family cohesion. Models 1 and 2 of the regression analysis further supported this result. According to family systems theory, different family members may perceive the same family environment differently [15]. Previous studies have also confirmed this idea, but our findings are not entirely consistent with previous findings. According to the generational stake hypothesis, the different roles of parents and children influence their family perceptions, with parents being more invested in their children and perceiving higher family cohesion compared to their children [43]. J Carola Pérez et al. [44] also found that mothers reported higher levels of family cohesion than their adolescent children. However, in the context of dementia caregiving, our findings indicate that adult-child caregivers perceived higher family cohesion than spousal caregivers. This discrepancy may be associated with contextual factors, such as the children’s age, ethnicity, clinical status, and family integrity [45]. Research on perceived parent-child differences in Chinese families remains limited. In many family contexts, younger generations are commonly expected to assume caregiving responsibilities for their elders [46]. This normative expectation may partly explain why adult-child caregivers are more likely to actively take on the caregiving role for older adults with dementia. In contrast, spousal caregivers may regard caregiving as a marital obligation rather than a voluntary or proactive act [47]. Furthermore, studies have shown that a strong sense of intergenerational responsibility is positively associated with life satisfaction [48], which may be linked to higher levels of perceived family cohesion among adult-child caregivers. From the perspective of family systems theory, the concept of triangulation provides additional insight. Triangulation refers to when the relationship between two people (husband and wife) faces tension or instability, the goal is shifted to a third party (children) to alleviate the conflict [15]. Thus, in this process, children may serve as emotional buffers, forming strong bonds with both parents to reduce familial tension. This pattern may be associated with a compensatory sense of emotional closeness perceived by the children [49], whereas the emotionally distant spouses may perceive lower levels of family cohesion. Gao et al. [50] described a similar pattern in their analysis of family typologies, identifying a “compensatory family” structure in which adult-children report high levels of family cohesion despite emotional detachment between the parents. In such families, mothers may seek deeper emotional connections with their children to compensate for marital dissatisfaction, further reinforcing the adult-children’s perception of family cohesion.

Another objective of this study was to examine factors associated with perceived family cohesion and to assess indirect associations between caregiver role (Factor A) and perceived family cohesion (Factor X) through caregiver-related characteristics (Factor B and Factor C). Caregivers’ self-reported physical health, as a caregiver’s personal resource, was found to mediate the relationship between caregiver role and perceived family cohesion. Specifically, adult-child caregivers exhibited better physical health and reported higher levels of perceived family cohesion compared to spousal caregivers. This may be associated with age-related differences between caregiver groups, as adult-child caregivers tend to be younger and in better physical condition. With advancing age, individuals are more likely to develop chronic conditions such as cardiovascular disease, cancer, and diabetes [51], which are associated with poorer overall health status. Moreover, caring for a person with dementia is widely recognized as highly demanding [5], and poorer physical health may be associated with reduced capacity to cope with caregiving demands. In contrast, caregivers in better physical condition are less likely to experience psychological distress, which may help reduce family conflict and enhance family cohesion [52]. In terms of family resources, the number of family members assisting in caregiving was greater among adult-child caregivers, and this was positively associated with higher levels of perceived family cohesion. This finding is consistent with previous studies. Although primary family caregivers are the main providers of care for older adults with dementia, support from other family members represents an important protective resource in the caregiving context. Insufficient family support has been linked to higher levels of caregiver burnout [53]. Therefore, dementia caregiving is a task that strongly depends on collaboration within the family. Adult-child caregivers may be more likely to coordinate caregiving tasks with spouses, siblings, and other relatives. In an intervention trial involving family caregivers of people with dementia, caregivers reported more positive interactions with family members and stronger feelings of connectedness, which were associated with improved perceptions of overall family relationships [54]. In terms of social resources, this study found that adult-child caregivers reported higher levels of social support compared to spousal caregivers. Similarly, Wang et al. [55] compared spousal and adult-child caregivers of Chinese dementia patients and found that spousal caregivers had weaker social networks compared to adult-child caregivers. Furthermore, this study found that higher levels of social support were significantly associated with stronger perceived family cohesion. Perceived social support is widely recognized as an important correlate of family functioning. Coping, hope, resilience, and optimism may serve as mediators between social support and family functioning [56]. Another study found that social support is associated with increased positive emotions and reduced negative emotional states among caregivers [57]. When caregivers feel supported and understood, they are more likely to engage in positive interactions, thereby strengthening emotional bonds and improving perceived family cohesion.

The perception and evaluation of stress (Factor C) is another critical variable that is associated with perceived family cohesion. In this study, Factor C was operationalized as caregivers’ self-efficacy. Our study demonstrated that self-efficacy was associated with perceived family cohesion and mediated the relationship between caregiver role and perceived family cohesion. Self-efficacy refers to an individual’s self-regulatory ability to respond to events, which is a judgment of one’s ability and confidence that one can achieve a particular goal. According to self-efficacy theory, self-efficacy is enhanced by the success of an individual’s behavior (direct experience) and the success of others (indirect experience) [28]. Adult-children may be more likely to actively seek out caregiving knowledge and experiences of caring for a person with dementia [46]. Moreover, since dementia typically progresses gradually [58], adult-child caregivers often assume caregiving responsibilities progressively as their parents’ condition deteriorates, allowing them to accumulate relevant experience over time. Compared to spousal caregivers, adult-child caregivers tend to have broader social networks and are more likely to engage in discussions about caregiving strategies with others [55], which may be associated with higher levels of self-efficacy through indirect experience. On the other hand, adult-child caregivers with higher self-efficacy perceived higher family cohesion. According to Lazarus and Folkman’s stress-coping theory [59], individuals’ responses to stress are shaped by their cognitive assessment of the situation. Those with high self-efficacy tend to confront challenges with the belief that they can overcome them through personal effort [28]. Previous research has consistently shown that higher self-efficacy is associated with lower perceived stress levels [29]. Therefore, adult-child caregivers with greater self-efficacy may be more confident in managing communication and coordination among family members, particularly in situations where caregiving responsibilities might otherwise undermine family cohesion. This pattern is associated with stronger perceptions of emotional connectedness within the family.

### Strengths and Limitations

This study, based on the ABC-X model, provides valuable insights into the relationship among caregiver role, caregiver-related characteristics, and perceived family cohesion. It thereby expands existing research on family caregiving and perceived family cohesion within dementia care contexts. To our knowledge, this is the first study to directly compare perceived family cohesion between spousal and adult-child caregivers, thereby offering new empirical evidence on family dynamics within caregiving contexts. Another strength is the inclusion of multiple caregiver-related characteristics, which provides a more comprehensive understanding of factors associated with perceived family cohesion.

Several limitations of this study need to be acknowledged. First, the use of cross-sectional data restricts the ability to establish causal relationships between core variables. Second, due to limitations in the survey design, this study lacked objective measures of caregivers’ physical health status and the severity of the patients’ dementia. Similarly, family resources were operationalized solely as the quantity of available support. While these indicators have been used in prior research, they may not fully capture the complexity of health status and family resources. Third, mediation analyses were conducted separately without correction for multiple comparisons, which may increase the risk of Type I error.

Future research should consider longitudinal and cross-cultural designs, incorporate objective biomedical indicators, and adopt comprehensive measures of family resources to enhance the robustness and generalizability of findings.

## 5. Conclusions

This study examined differences in perceived family cohesion across caregiver roles and, based on the ABC-X model, found that adult-child caregivers reported higher levels of perceived family cohesion than spousal caregivers. Caregiver-related characteristics mediate the relationship between caregiver role and perceived family cohesion. These findings should be interpreted within the limitations of the cross-sectional design and specific caregiving context.

Given that caregiver-related characteristics act as mediators, interventions should focus on strengthening caregivers’ physical health, social support, self-efficacy, and family involvement in caregiving to equalize the perception of family cohesion between spousal caregivers and adult-child caregivers. The findings suggest that family-based support strategies may prove more effective when tailored to the caregiver role. For example, interventions targeting spousal caregivers could prioritize strengthening social connections, mobilizing family support, and enhancing caregivers’ confidence in managing caregiving challenges. For adult-child caregivers, maintaining existing family coordination and support networks may help sustain perceived family cohesion.

By focusing on perceived family cohesion, this study contributes to the dementia care research literature. It extends existing research scope, emphasizing the importance of family relational outcomes in dementia care. It provides evidence-based insights for developing role-sensitive, family-based support services in dementia care, while acknowledging the need for longitudinal research to further clarify the direction and dynamics of these associations.

## Figures and Tables

**Table 1:** Background Information of Participants.

Variables	Category	Spousal (N = 154)	Adult-Child (N = 256)	Total (N = 410)	*p*
		N (%)	N (%)	N (%)	
Age	<60	3 (1.95)	121 (47.27)	124 (30.24)	<0.001
	60~79	74 (48.05)	133 (51.95)	207 (50.49)	
	≥80	77 (50.00)	2 (0.78)	79 (19.27)	
Sex	Male	61 (39.61)	148 (57.81)	209 (50.98)	<0.001
	Female	93 (60.39)	108 (42.19)	201 (49.02)	
Employment status	Yes	1 (0.65)	74 (28.91)	75 (18.29)	<0.001
	No	153 (99.35)	182 (71.09)	335 (81.71)	
Daily caregiving hours	<10	17 (11.04)	83 (32.42)	100 (24.39)	<0.001
	10~19	14 (9.09)	49 (19.14)	63 (15.37)	
	≥20	123 (79.87)	124 (48.44)	247 (60.24)	
Years since patient diagnosis	<5	74 (48.05)	142 (55.47)	216 (52.68)	0.173
	5~9	41 (26.62)	68 (26.56)	109 (26.59)	
	≥10	39 (25.32)	46 (17.97)	85 (20.73)	
Contracted with a family doctor	Yes	6 (3.90)	17 (6.64)	23 (5.61)	0.242
	No	148 (96.10)	239 (93.36)	387 (94.39)	

**Table 2:** Perceived Family Cohesion Between Spousal Caregivers and Adult-Child caregivers.

Category	Spousal (N = 154)	Adult-Child (N = 256)	*p*
	N (%)/M (SD)	N (%)/M (SD)	
Total score	60.64 (11.79)	66.06 (10.05)	<0.001
Disengaged	51 (33.12)	43 (16.80)	<0.001
Separated	38 (24.68)	54 (21.09)	
Connected	35 (22.73)	81 (31.64)	
Enmeshed	30 (19.48)	78 (30.47)	

**Table 3:** Results of Multiple Linear Regression Analysis on Factors Influencing Perceived Family Cohesion.

Variables	Model 1			Model 2			Model 3		
	B	SE	β	B	SE	β	B	SE	β
(constant)	60.636 ***	0.865		47.216 ***	5.957		17.329 **	6.202	
Role (ref. = spousal)	5.422 ***	1.095	0.238	6.796 ***	1.686	0.299	3.148 *	1.584	0.138
Age				0.155 *	0.07	0.181	0.185 **	0.063	0.215
Sex (ref. = male)				0.365	1.111	0.017	0.345	1.003	0.016
Daily caregiving hours				−0.102	0.07	−0.076	0.023	0.065	0.017
Years since patient diagnosis				−0.207 *	0.09	−0.109	−0.152	0.08	−0.08
Contracted with a family doctor (ref. = NO)				6.945 **	2.298	0.145	6.683 **	2.038	0.139
Employment status (ref. = employed)				−2.618	1.722	−0.092	−2.489	1.531	−0.087
Self-reported physical health							1.297 *	0.533	0.111
Social support							0.561 ***	0.068	0.392
Self-efficacy							0.151 *	0.068	0.1
Family members assisting							1.071 *	0.529	0.098
F	24.533 ***			7.069 ***			16.196 ***		
R^2^ (Adjusted R^2^)	0.057 (0.054)			0.110 (0.094)			0.309 (0.290)		

Note: B: Unstandardized coefficients, SE: Standard error, β: Standardized coefficients; * *p* < 0.05, ** *p* < 0.01, *** *p* < 0.001.

**Table 4:** Regression Coefficients in the Mediation Analysis.

Mediator	Result Variable	Predictor Variable	R	R^2^	F	β
Personal resource (Factor B-a)	Physical health	Role	0.297	0.088	39.530 ***	0.613 ***
	Family cohesion	Role	0.292	0.085	18.981 ***	0.383 ***
		Physical health				0.177 ***
Family resource (Factor B-b)	Family members assisting	Role	0.428	0.183	91.413 ***	0.882 ***
	Family cohesion	Role	0.297	0.088	19.718 ***	0.318 **
		Family members assisting				0.197 ***
Social resource (Factor B-c)	Social support	Role	0.316	0.100	45.300 ***	0.652 ***
	Family cohesion	Role	0.489	0.239	63.885 ***	0.198 *
		Social support				0.450 ***
Self-efficacy (Factor C)	Self-efficacy	Role	0.208	0.043	18.440 ***	0.429 ***
	Family cohesion	Role	0.310	0.096	21.600 ***	0.404 ***
		Self-efficacy				0.203 ***

Note: β: Standardized coefficients; * *p* < 0.05, ** *p* < 0.01, *** *p* < 0.001.

**Table 5:** Results of the Mediation Analysis.

Path	Effect	Boot SE	Boot LLCI	Boot ULCI	Ratio
1. X → Personal resource → Y	0.109	0.034	0.048	0.182	22.2%
2. X → Family resource → Y	0.174	0.049	0.082	0.277	35.4%
3. X → Social resource → Y	0.293	0.049	0.200	0.391	59.7%
4. X → Self-efficacy → Y	0.087	0.031	0.034	0.154	17.7%

Note: Standardized indirect effect of X on Y is shown. The bootstrap 95% CIs do not contain zero.

## Data Availability

The data presented in this study are available on request from the corresponding author due to ethical restrictions and the need to protect participant privacy.
